# Electroporation and cell killing by milli- to nanosecond pulses and avoiding neuromuscular stimulation in cancer ablation

**DOI:** 10.1038/s41598-022-04868-x

**Published:** 2022-02-02

**Authors:** Emily Gudvangen, Vitalii Kim, Vitalij Novickij, Federico Battista, Andrei G. Pakhomov

**Affiliations:** 1 Frank Reidy Research Center for Bioelectrics, Old Dominion University, 4211 Monarch Way, Room 340, Norfolk, VA 23508 USA; 2 Vilnius Gediminas Technical University, Vilnius, Lithuania; 3 Department of Information Engineering, Electronics and Telecommunications, Sapienza University of Rome, Rome, Italy

**Keywords:** Cancer therapy, Cell death

## Abstract

Ablation therapies aim at eradication of tumors with minimal impact on surrounding healthy tissues. Conventional pulsed electric field (PEF) treatments cause pain and muscle contractions far beyond the ablation area. The ongoing quest is to identify PEF parameters efficient at ablation but not at stimulation. We measured electroporation and cell killing thresholds for 150 ns–1 ms PEF, uni- and bipolar, delivered in 10- to 300-pulse trains at up to 1 MHz rates. Monolayers of murine colon carcinoma cells exposed to PEF were stained with YO-PRO-1 dye to detect electroporation. In 2–4 h, dead cells were labeled with propidium. Electroporation and cell death thresholds determined by matching the stained areas to the electric field intensity were compared to nerve excitation thresholds (Kim et al. in Int J Mol Sci 22(13):7051, 2021). The minimum fourfold ratio of cell killing and stimulation thresholds was achieved with bipolar nanosecond PEF (nsPEF), a sheer benefit over a 500-fold ratio for conventional 100-µs PEF. Increasing the bipolar nsPEF frequency up to 100 kHz within 10-pulse bursts increased ablation thresholds by < 20%. Restricting such bursts to the refractory period after nerve excitation will minimize the number of neuromuscular reactions while maintaining the ablation efficiency and avoiding heating.

## Introduction

Tumor ablation by pulsed electric fields (PEF) is a fast-growing field with its distinct niche in the arsenal of anti-cancer treatments. This method relies on irreversible electroporation (IRE), which results in a severe disruption of the membrane barrier of tumor cells leading to irreversible changes in homeostasis and cell death.

IRE treatments utilize pulses of a single polarity, typically about 100 µs in duration. Pulse number and amplitude are set to cause lethal cell injuries, while concurrent Joule heating is contained below the point of thermal damage. IRE ablation destroys the cellular component of tissues while sparing its architecture including the collagen scaffold, renal collecting system, and vascular and ductal structures^1^. Such selectivity of PEF ablation facilitates the orderly repopulation of treated areas by healthy cells and functional recovery. Preclinical and clinical data point out that IRE ablation is safe, efficient, and promising for elimination of tumors located in thermally sensitive organs with high vascularity and structural density, such as pancreas, liver, prostate, and kidney^1–12^. IRE is increasingly used for the ablation of deep-seated tumors, inoperable tumors, and lesions unresponsive to alternative treatments.

Many benefits of IRE ablation are offset by concurrent neuromuscular excitation, which includes severe pain and involuntary muscle contractions^12–16^. For 100-µs pulses, nerve excitation occurs at the electric field strength three orders of magnitude lower than what is required for IRE, causing nerve excitation tenfold farther away from electrodes^17^. While local and/or general anesthesia and muscle relaxants offer a partial solution to the neurostimulation problem, the optimal solution would be to minimize nerve excitation in the first place. IRE treatments will be far more attractive and gain broader recognition by medical practitioners if the ablation could be accomplished with minimal or no neuromuscular side effects. This goal can potentially be accomplished by choosing the proper PEF parameters, such as the pulse duration, shape, and repetition rate.

A number of in vitro studies found that short nanosecond pulses (nsPEF) have relatively low efficiency for stimulation of excitable cells and tissues^18–23^. Paradoxically, electroporation took place at the electric field strengths lower than the threshold to evoke action potentials. Instead of a “direct” excitation by membrane depolarization, nsPEF caused electroporation that led to the loss of the resting membrane potential and elicited action potentials as a downstream effect of membrane disruption^18–20^. The convergence of the electroporation and excitation thresholds is possibly explained by a minimum time interval needed for the opening of voltage-gated ion channels and whether the transmembrane potential can be retained above the threshold long enough after nsPEF^24^. These findings suggested that nsPEF is less efficient for stimulation than for electroporation and tissue ablation.

It has long been known that nerve stimulation efficiency of short duration PEF can be further reduced by switching the pulse polarity^25–30^. As a result, bipolar nsPEF delivers twofold more energy but is less efficient at nerve excitation than a single phase of the same PEF. However, bipolar nsPEF are also less efficient at electroporation and cell killing^23,31–36^. The mechanism of this “bipolar cancellation” phenomenon has not been fully understood. It remains to be tested if bipolar nsPEF suppress neurostimulation stronger than electroporation, thereby offering an additional benefit for ablation treatments.

An independent quest for IRE with low neuromuscular side effects has resulted in the introduction of a high-frequency irreversible electroporation ablation (H-FIRE)^9–11,17,37,38^. This modality utilizes complex protocols of brief bipolar pulses (down to 0.5–2 µs) to destroy tumors. Both model computations and numerous experimental studies have demonstrated successful tumor ablations by H-FIRE while the neuromuscular stimulation was markedly reduced. H-FIRE showed efficiency for melanoma, carcinoma, glioblastoma, meningioma, hepatoma and other cancer types. Benefits of H-FIRE have now been proven beyond doubt and explored even more thoroughly than with simpler short-pulse ablation protocols. In fact, neuromuscular effects can be also markedly reduced with unipolar nsPEF delivered at a low frequency^39^. Applying nsPEF at low repetition rates or splitting pulse trains into short bursts with long intervals alleviates the concerns for concurrent thermal effects and may have an additional benefit of ablation with fewer pulses, by inducing electrosensitization^40–42^.

We have recently quantified the impact of the electrode configuration, PEF strength (up to 20 kV/cm), repetition rate (up to 3 MHz), bi- and triphasic pulse shapes, and pulse duration (10 ns to 1 ms) on the excitation threshold for fast peripheral nerve fibers^43^. The thresholds for single unipolar but not bipolar stimuli followed the classic strength–duration dependence. Symmetrical bipolar nsPEF required a sharply higher electric field to cause excitation than unipolar and asymmetrical stimuli. We also found that the temporal summation effect for repetitive nsPEF above the critical duty cycle of 0.1% is far weaker for bipolar than for unipolar nsPEF.

The results reported below are the second part of this study, which was aimed at the quantification of cell death and electroporation thresholds for a matching selection of PEF exposure conditions. The overarching aim of this work was to compare the experimentally measured electroporation and excitation thresholds to identify PEF protocols most efficient at cell killing and least efficient at neurostimulation. We also considered such potentially important exposure characteristics as the absorbed dose and the size of the reversible electroporation fringe. In contrast to neurostimulation, cell killing at achievable electric field strengths requires the delivery of multiple pulses, so the respective number of neuromuscular responses evoked in the process of ablation was taken into account as well. Overall, our results came in agreement with previous reports that the minimum ratio of the ablation and excitation thresholds is achieved with symmetrical bipolar nsPEF, with the best result for the shortest pulses tested (150 ns). The concurrent increase of the absorbed dose and potential thermal effects can be mitigated by packing nsPEF in brief bursts. Limiting duration of these bursts to nerve refractory period helps to further restrict the neuromuscular response.

## Methods

### Cell line and propagation

Murine colon carcinoma cells purchased from the American Type Culture Collection (ATCC, Manassas, VA, CRL-2638) were maintained at 37 °C, 5% CO_2_ in RPMI-1640 medium with 25 mM HEPES (Cat. # MT10041CV, Thermo Fisher Scientific, Waltham, MA). The medium was supplemented with 10% fetal bovine serum (Atlanta Biologicals, Norcross, GA), 100 I.U./ml penicillin, and 0.1 μg/ml streptomycin (GIBCO, Gaithersburg, MD). High HEPES content medium was chosen to mitigate pH changes while outside of the incubator as well as possible pH changes due to PEF exposure^44^. Cells were seeded on 24-well plates (μ-Plate 24 Well Black, IBIDI, Gräfelfing, Germany) and grown to 80–90% confluency over 3–5 days, refreshing medium as needed.

### Electroporation of cells in monolayers and defining the electric field thresholds

Electroporation was accomplished by applying electric pulses between two cylindrical electrodes inserted vertically into the wells and gently brought in touch with the monolayer. These electrodes produced a non-uniform electric field decaying gradually with the distance from the electrodes. This approach enables labor-efficient testing of a wide range of the electric field strengths in a single PEF-exposed sample ^17,31,45–48^. Electroporated and dead cells are identified by the uptake of cell-impermeable dyes, and the respective thresholds are measured by matching the margins of the stained area to the local electric field strength (see below for details).

### PEF generators

**Figure 1:**
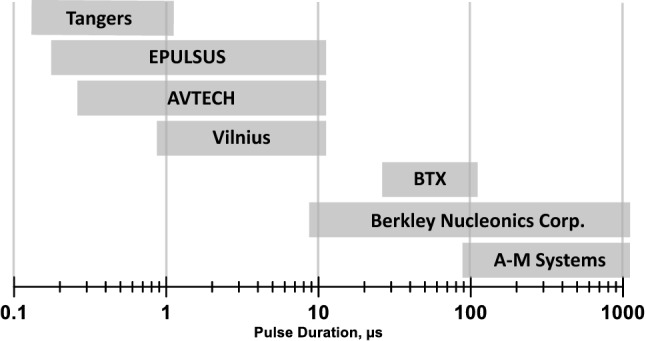
The range of pulse durations covered by different generators employed in this study. See text for more details.

Multiple pulse generators were employed to electroporate cells by pulses from 1 ms down to 150 ns in duration, from 25 V up to 5.6 kV in amplitude, at up to 1 MHz repetition rates, with uni- and bipolar pulse shapes (Fig. 1). Seven pulse generators were used to deliver unipolar pulses: the Model 4100 isolated high-power stimulator (A-M systems, Carlsborg, WA), the Model 6040 mainframe with a 202H 300V insert (Berkley Nucleonics Corporation, San Rafael, CA), the BTX ECM830 (Harvard Bioscience, Inc., Holliston, MA), the AVTECH AVOZ-D2-B-ODA (AVTECH Electrosystems, Ottawa, Ontario, Canada)^49^, two custom-built generators (Tangers Electronics, Norfolk^40^, and VA Vilnius Gediminas Technical University, Vilnius, Lithuania^50^), and the EPULSUS-FPM4-7 (Energy Pulse Systems, Lisbon, Portugal). Unless specified otherwise below, we applied trains of 10, 100, or 300 pulses at 10 Hz. Pulse shape and amplitude were continuously monitored with a TDS 3052 oscilloscope (Tektronix, Beaverton, OR, USA).

The EPULSUS generator was employed to produce a bipolar electric field, by alternating the energized electrode (anode) with the ground electrode^43^. Studies with bipolar PEF were limited to short pulses (≤ 10 µs) which are expected to evoke the bipolar cancellation effect ^25,32,43^. The duration and amplitude of the second phase were always set equal to the first phase. The amplitude and duration of bipolar pulses reported in this paper are those of the first phase (the peak-to-peak amplitude and the total pulse duration are twofold larger).

### Electrodes and automated electrode positioning

**Figure 2:**
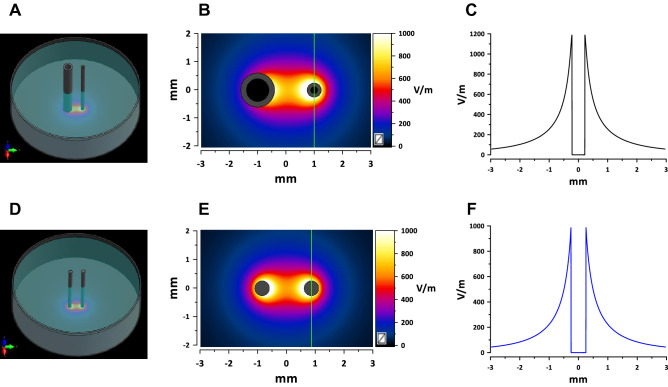
Electric field simulations for the asymmetrical (**A**–**C**) and symmetrical **(D**–**F)** electrode assemblies. (**A**) and (**D**) show the spatial configuration of the electrode assemblies orthogonal to a plastic dish and touching its bottom. (**B**) and (**E**) show the calculated electric field distribution in a plane 5 µm above the bottom with 1 V applied between electrodes. The direction in which the electric field thresholds were measured is marked by vertical green lines (see text). (**C**) and (**F**) are the electric field values along the green lines. These values were linearly scaled to the voltage applied between the electrodes for calculation of the electric field thresholds.

Preliminary experiments showed that trains of “long” pulses (≥ 100 µs) at lethal intensities caused intense bubble formation at the cathode. The bubbles covering the electrode surface could unpredictably change the electric field distribution during a pulse train. Increasing the cathode diameter reduced the electric field strength at its surface and eliminated bubble formation. In sets of experiments which included the “long” pulses, we used an asymmetrical electrode assembly (Fig. 2A) for all pulse durations. The electrodes were made of stainless steel hollow rods, 1.2 mm outer diameter for cathode and 0.5 mm for anode. They were placed 2.2 mm apart (center-to-center), parallel to each other and orthogonal to the cell monolayer, in a custom-built holder that prevented downward advancement of the electrodes once they reached the well bottom.

Experiments with bipolar waveforms and with different pulse repetition rates were limited to pulse durations of 10 µs or less. In these sets of experiments, we used a symmetrical electrode assembly (Fig. 2D) of two parallel tungsten rods 0.5 mm in diameter at 1.7-mm distance (center-to-center). Some exposure conditions were tested with both the symmetrical and asymmetrical electrode assemblies, yielding similar PEF threshold measurements (data not shown).

The electrode holder was mounted in a customized Anet A8 3D printer (Shenzhen Anet Technology Co, China) in place of the extruder. A 24-well plate with cells was fixed firmly in a frame attached to the printer stage in a precise position relative to the electrodes. The printer was programmed to deliver electrodes exactly to the center of the first well, stay put for 60 s for PEF exposure, then move to the second well, and so forth. The 60 s interval was kept constant even if PEF exposure took less time, to ensure a constant time interval between PEF treatments and image acquisitions (performed in the same sequence also taking 60 s per well).

### Experiment protocol, cell staining, and image acquisition

Fluorescent dyes Hoechst-33342, YO-PRO-1 (YP), and propidium iodide (Pr) were obtained from Thermo Fisher Scientific (Waltham, MA) and added to the complete growth medium when needed at 2.25, 1, and 75 µM, respectively.

PEF exposures, subsequent manipulations, and imaging were performed with cells in the complete growth medium. Shortly prior to the onset of PEF exposures, Hoechst and YP dyes were added to the medium in the wells. Cell-permeable Hoechst dye labeled the nuclei of all cells and helped to identify gaps and nonuniformities in the cell monolayer. YP has poor permeability into intact cells and was used to label cells with membrane integrity compromised by electroporation, including nanoelectroporation^31,32,35,48,51–53^. Cells were rinsed with a fresh growth medium without phenol red to remove the dyes 30 min after PEF exposure and the plate was returned into a CO_2_ incubator.

**Figure 3:**
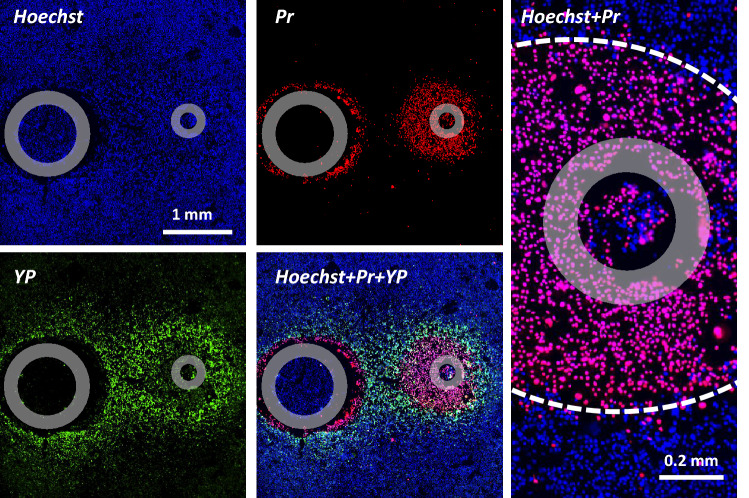
An example of fluorescent staining of cell monolayer for the determination of electric field thresholds for electroporation and cell death. Cells were exposed to 300 unipolar pulses (50 µs duration, 10 Hz, 150 V). Individual panels show the signal from Hoechst-33342 (***Hoechst***), YO-PRO-1 (***YP***), and propidium iodide (***Pr***) dyes, as well as their combinations as marked in the legends. Gray circles mark the footprints of the electrodes. Right panel shows the area near the anode at a higher magnification and the margins of the cell death area marked by a dashed white line. Note peeling of the monolayer near the cathode and quenching of YP signal by Pr. See text for more details.

Pr was added to the samples in 2 or 4 h after PEF exposure, to label cells that had failed to restore the membrane integrity (in Section "The time course of cell death following PEF treatments", Pr was added at intervals from 0.1 to 24 h after PEF). In 10 min, the plate was transferred to an IX83 microscope (Olympus America, Hamden, CT) custom configured with an automated MS-2000 scanning stage (ASI, Eugene, OR), X-Cite 110LED illuminator (Excelitas Technologies Corporation, Waltham, MA) and an ORCA-Flash4 sCMOS camera (Hamamatsu, Shizuoka, Japan). A total of 9 images of adjacent regions (3 × 3 square) were taken with a 10x, 0.38 NA objective and stitched into one high-resolution image. The samples were imaged with DAPI, FITC and Cy3 filter sets for Hoechst, YP, and Pr signals, respectively (Fig. 3). Pr expectedly quenched the YP signal in Pr-permeable (presumably dead) cells^48,54^. The image acquisition, including the filter cube selection, stage re-positioning and synchronization with illumination and camera operation, were accomplished automatically with CellSens software (Olympus).

We used YP uptake as a convenient and sensitive index of electroporation of cells in a monolayer, and the delayed Pr uptake as a sign of cell death^48^.Most Pr-positive cells displayed full quenching of YP signal by Pr and strong Pr fluorescence, indicative of an unrestricted Pr entry. This “all-or-none” Pr uptake pattern enabled a fairly straightforward identification of the margins of the area where the electric field was strong enough to cause cell death. This area encompassed the electrode(s), whose position on the image was identified by the footprint(s). The distance from the margin of the cell death area to the center of the electrode (the smaller electrode only for the asymmetrical configuration or both electrodes for the symmetrical one) was measured in the direction perpendicular to the line that connects the electrodes (marked by vertical green lines in Fig. 2B,E). The respective electric field threshold was calculated as the simulated electric field multiplied by the applied voltage (Fig. 2 and Section "Electric field simulations"). The voltage appropriate to produce a measurable lesion (ideally within 0.3–1 mm from the electrode) was determined in preliminary experiments for each pulse and train duration. On many occasions, the electric field thresholds were measured with more than one applied voltage; these experiments produced similar threshold values which were pooled together for analyses.

In contrast to the ”all-or-none” Pr staining, the degree of YP uptake decreased with the field strength, eventually blending into the background away from the electrodes. The margins of the reversible electroporation could not be uniquely established and would depend on the YP uptake assumed as the threshold. We have arbitrarily chosen to put the margin of electroporation where the YP fluorescence intensity exceeded the background by 20%. The intensity was measured with ImageJ Fiji platform^55^ in the direction marked by vertical green lines in Fig. 2B,E. The fluorescence intensity leveled off to a plateau away from the electrode(s), and this plateau intensity was taken as a background for each individual sample (this background value was a sum of spontaneous YP uptake, autofluorescence, and measurement noise). Subsequent calculations were the same as for the Pr threshold.

### Thermometry

**Figure 4:**
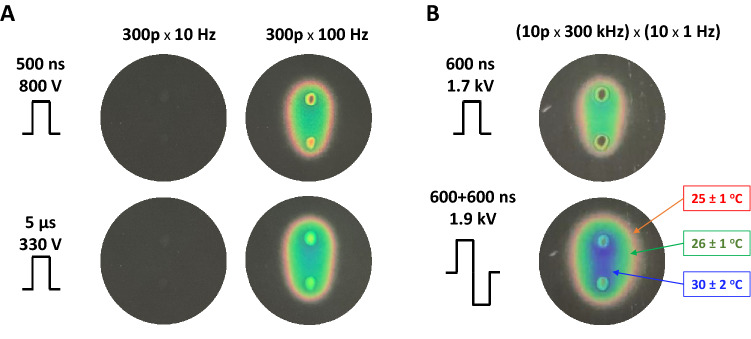
Monitoring the temperature rise due to PEF exposure with thermochromic liquid crystal sheets. Images were taken by the end of exposure to a train of 300 pulses at 10 or 100 Hz (**A**) or to a series of 10 bursts at 1 Hz (**B**). Each burst consisted of 10 uni- or bipolar pulses at 300 kHz. Other exposure parameters are marked in the legends. Temperatures at the color transitions are labeled in panel B according to the manufacturer’s specifications.

Maximum heating from PEF treatments was assessed with R25C5B calibrated thermochromic liquid crystal sheets (LCR Hallcrest, Glenview, IL). Sheets were placed on the bottom of the well, in contact with electrodes, and color changes were recorded with a digital camera^48^. Measurements were performed at the room temperature of 22–23 °C. Pulse amplitude was set to the maximum that was used for specific pulse durations in biological experiments. No color changes were observed when a train of 300 pulses was applied at 10 Hz, which is the repetition rate that was used in most experiments. With the repetition rate increased to 100 Hz (as a positive control), the color changed to red (25 ± 1 °C) and green (26 ± 1 °C) with just a hint of transition to blue (30 ± 2 °C), Fig. 4A.

Experiments aimed at understanding frequency effects of PEF (Section "Electroporation and cell killing by a series of nsPEF bursts: The effect of pulse repetition rate within the bursts") utilized uni- and bipolar 600-ns pulses at 1.7 and 1.9 kV. A total of 100 pulses were applied as 10 bursts of 10 pulses each. The frequency was varied within the bursts (up to 1 MHz), while the bursts were delivered at a constant rate of 1 Hz, to allow time for heat dissipation. Nonetheless, such exposures caused significant heating (Fig. 4B). By the end of exposure, the temperature remained below the green–blue transition at 30 ± 2 °C for unipolar pulses but exceeded it for bipolar pulses. The regions where we measured the thresholds of YP and Pr uptake were either in the green-colored region or close to the transition from green to blue. The next color transition (blue to black, at 44 ± 2 °C) was not observed.

Overall, the temperature measurements established that heating was not a considerable factor, except for the experiments in Section "Electroporation and cell killing by a series of nsPEF bursts: The effect of pulse repetition rate within the bursts" where the combined effects of electroporation and heating could not be ruled out.

### Electric field simulations

The electric field was calculated with a low-frequency finite element solver Sim4Life V5.2 (Zurich Med Tech, Zurich, Switzerland). The asymmetrical electrode array was modeled as two parallel hollow rods with outer/inner diameters of 1.2/0.8 mm and 0.5/0.25 mm, at 2.2 mm center-to-center distance (Fig. 2A). The symmetrical array was modeled as two solid rods 0.5 mm in diameter at 1.7 mm center-to-center distance (Fig. 2D). The electrodes were positioned perpendicular to and touching the bottom of a 16.3-mm diameter plastic well filled to a 2.3-mm height with a 1.4 S/m solution. The electric field values used for threshold calculations throughout this study and shown in Fig. 2 are those measured in the plane orthogonal to the electrodes, at the height of 5 µm above the bottom.

### Statistical analysis

**Figure 5:**
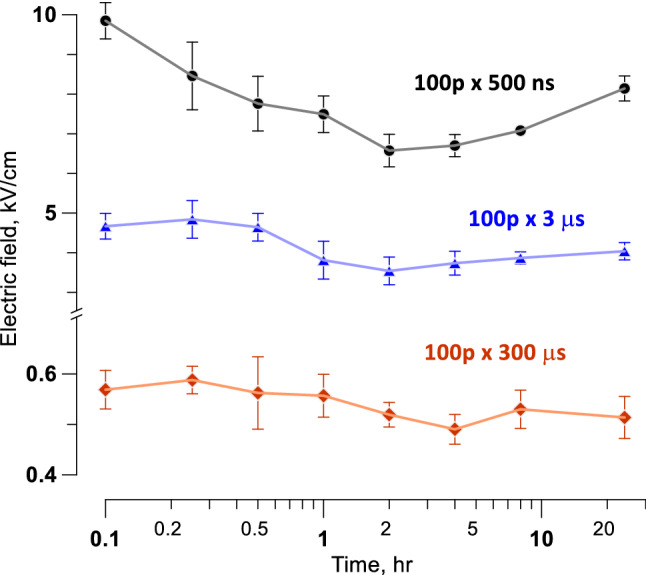
Electric field thresholds for cell death as measured at different time intervals after PEF exposure (100 pulses at 10 Hz; pulse duration is marked in the legends). The smallest thresholds were measured at 2 and 4 h after the exposure. See text for details.

**Figure 6:**
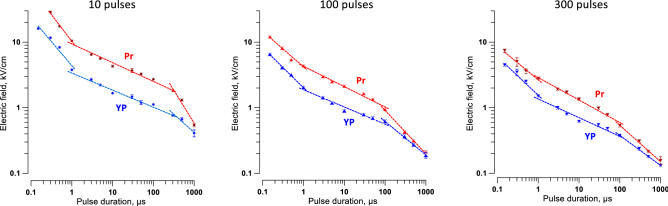
Electric field thresholds for electroporation (YP) and cell killing (Pr) by unipolar pulses of different duration. Trains of 10, 100, or 300 pulses (left to right panels) were applied at 10 Hz. Electroporation thresholds were measured from the borders of YO-PRO-1 (YP) dye uptake; the dye was added before the pulse treatment and removed 30 min after it. Electric field thresholds for cell death were measured from the borders of propidium (Pr) staining when the dye was added 4 h after the pulse treatment. See text for more details. Dashed lines are the best fits using power function, separately for pulse duration less than 1 µs; from 1 µs to 100 or 300 µs; and for still longer pulses. Error bars may be not visible when they are smaller than the central symbol.

**Figure 7:**
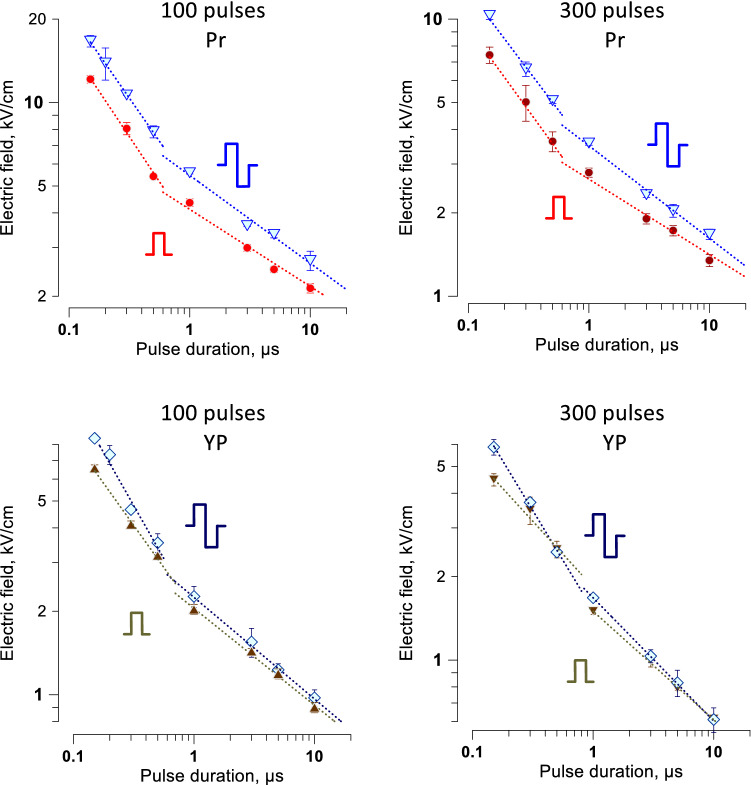
The addition of the opposite polarity second phase increases the threshold for cell killing and electroporation. The thresholds were measured by the margins of Pr and YP uptake and are presented at the top and bottom panels, respectively (see Fig. 6 and text for details). The data for unipolar PEF are the same as in Fig. 6. Cells were exposed to trains of 100 or 300 pulses at 10 Hz. For bipolar pulses, the duration of a single phase was used in lieu of the total pulse duration. Note that the difference in thresholds becomes larger for shorter pulses. Differences between uni- and bipolar pulses for Pr uptake thresholds were statistically significant for 12 out of the 14 datapoints. Differences in YP uptake were significant only for the shortest pulses (two-tailed *t* test at p < 0.05).

**Figure 8:**
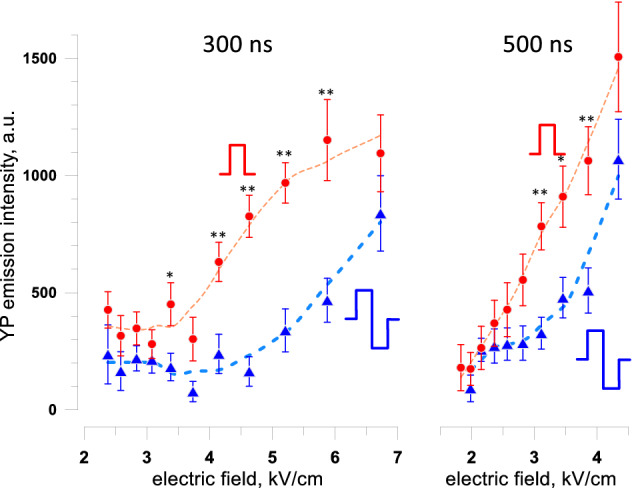
The addition of the opposite polarity second phase inhibits the electroporative uptake of YO-PRO-1 dye (YP) in a broad range of supra-threshold electric field strengths. Measurements were performed in cell samples exposed to 300-pulse trains at 10 Hz. Unipolar pulse duration and one phase duration of bipolar pulses was 300 ns (left panel) or 500 ns (right panel). YP was added to cell samples before pulse exposure and washed away 30 min after it. Dashed lines are the LOESS function best fits. * p < 0.05, ** p < 0.01 for the difference between bipolar and unipolar pulses, with two-tailed *t* test. See text for more details.

**Figure 9:**
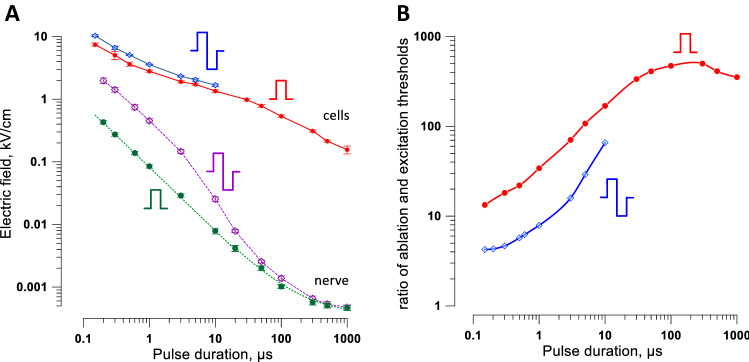
A comparison of the electric field thresholds for cell killing and for peripheral nerve stimulation. (**A**) Strength-duration curves for cell killing (solid lines) and for nerve excitation (dashed lines). Cell killing data are for trains of 300 uni- or bipolar pulses at 10 Hz (same data as in Figs. 6 and 7). Nerve stimulation data are for a single uni- or bipolar pulse as measured in isolated sciatic nerves in a conductive medium (from Fig. 2C in Ref. ^43^). Error bars may be smaller than the central symbol. (**B**) Ratios of the cell killing and nerve stimulation thresholds plotted against pulse duration (for bipolar pulses, against one phase duration).

Within each series of experiments, different conditions were randomized and tested in different cell samples 5 to 10 times for most datapoints. One experiment delivered just a single measurement of the electric field threshold for Pr and YP uptake for a tested combination of pulse parameters. Therefore, each individual graph in Figs. 5, 6, 7, 8 and 9 uses the data from 50–60 to more than 100 independent experiments. Data fits were calculated from individual experimental measurements withing a specific range of pulse parameters, from 30 to over 100 independent experiments per fit. The data for one condition could be collected using different pulse voltages and different pulse generators (see section “Experiment protocol, cell staining, and image acquisition”), with no noticeable impact on the measured electric field thresholds. The data were pooled together and analyzed with a two-tailed *t*-test and non-parametric tests as applicable. Grapher 16 (Golden Software, Golden, CO) was used for graph preparation, data fitting, and calculations of fit quality and correlation coefficients. For some datapoints in the graphs, the error bars are smaller than the central symbol and may not be visible in the graphs. In most cases, the differences between studied conditions were large and statistically significant at *p* < 0.01 or better. With the large number of datapoints and the error bars far from overlapping, we labeled the significance only in some graphs, to minimize the use of special symbols and preserve clarity.

## Results

### The time course of cell death following PEF treatments

Cell death can occur with an interval of hours after electroporation^56,57^. These preliminary experiments were aimed at determining the optimal time to measure the electric field thresholds for cell death in the cell monolayer model of PEF exposure.

Cell monolayers were exposed to 100-pulse trains of 0.5-, 3-, or 300-µs PEF at 10 Hz. Pulse amplitudes were set to produce a readily measurable cell death area of 0.5–1 mm in diameter. The border of this area was matched to the local electric field strength calculated for the chosen pulse amplitude. Cells were kept in the incubator and labeled by the addition of Pr at 0.1- to 24-h time intervals after PEF treatments. Samples were imaged at 10–15 min following Pr addition and discarded. With the shortest intervals, Pr could stain some transiently electroporated cells which would recover later on. However, the recovery was unlikely for longer intervals since most membrane repairs that can be accomplished are usually accomplished within 10–15 min after exposure^30,54^. Indeed, Pr staining was “all or none,” without intermediate staining for partially permeabilized live cells (Fig. 3).

For all tested PEF parameters, the largest area of Pr uptake (corresponding to the smallest threshold for cell killing) was measured at 2–4 h after the exposure (Fig. 5). For longer intervals, it either stayed the same or shrunk, due to the detachment of dead cells and repopulation of the area by survived cells. We have not observed any additional cell death at up to 24 h intervals. In all experiments described below, Pr was added at either 2 or 4 h after exposure, to measure the minimum electric field strength required to kill cells.

### Strength-duration curves for unipolar pulses from 150 ns to 1 ms

Pulse duration is the major parameter that determines the electric field thresholds for diverse biological effects, including nerve stimulation, electroporation, and cell death^17,37,43,52,57–61^. Most of the previous studies focused on the severity or probability of the effects rather than on the electric field thresholds and also were limited to relatively narrow PEF duration ranges.

We measured the electroporation and cell death thresholds by YP and Pr uptake, respectively, for pulses varying in duration from 150 ns to 1 ms (Fig. 6). They were applied in 10-, 100-, or 300-pulse trains at 10 Hz. The thresholds were smaller for longer trains and expectedly increased as the pulse duration decreased. This decrease followed a power function but was not monotonous as one would expect, and we were unable to fit the entire curve with any single function. The slope of the best fit using power function changed at two critical pulse durations, about 1 µs and 100 µs. When these “bends” were initially observed, they were thought to be an artifact from using different pulse generators or a random fluctuation due to a small number of experiments. However, adding more experiments and using different pulse generators only decreased the error bars and added confidence that the bends were real. Of note, some previous studies also observed an upward bend of the irreversible electroporation thresholds at about 1 µs^17,47^. The bends were similarly observed for all tested train lengths and may be indicative of different mechanisms of membrane permeabilization and cell death engaged by PEF.

The YP and Pr uptake showed strong correlation (R > 0.98) for all tested conditions, consistent with expectations that cell death is the consequence of electroporation. The shortest trains (10 pulses) had the largest difference in Pr and YP uptake thresholds (Fig. 6A), i.e., produced the largest fraction of reversibly electroporated cells. This feature is not desirable for ablation but may be useful in other applications such as electrochemotherapy and gene electrotransfer (also see Section "The balance of reversible and irreversible electroporation at different pulse durations"). The longest trains of 300 pulses killed cells at the lowest electric field strengths, making them potentially most promising for ablation with reduced neuromuscular stimulation.

### Strength-duration curves for bipolar pulses from 150 ns to 10 µs

The addition of the opposite polarity second phase to pulses longer than 10–50 µs has the same effect as simply the increase of the pulse width, i.e., it enhances biological effects and reduces their thresholds ^59^. In contrast, the addition of the opposite polarity phase to nsPEF evokes so-called “bipolar cancellation” that inhibits both electroporation^23,32–34,62^ and electrostimulation^25,43^. Stronger inhibition of the latter could offset neurostimulation effects of ablation treatments.

The increase of thresholds for electroporation and for cell death when applying bipolar instead of unipolar pulses was evaluated for 100- and 300-pulse trains (Fig. 7). The strength-duration curves had the same characteristic “bend” at about 1 µs as described above for unipolar pulses (Fig. 6). Bipolar cancellation increased the threshold for cell killing by the shortest 150-ns pulses just 1.3–1.4 times (*p* < 0.01). The difference in electroporation thresholds (YP uptake) between uni- and bipolar pulses was even smaller. Although the thresholds increased just marginally, the effects of bipolar pulses above the threshold were substantially weaker, and YP uptake by electroporated cells was reduced three–fivefold (Fig. 8). These results are consistent with earlier observations that bipolar cancellation inhibits electroporation but has little effect on the threshold electric field strength^35,48^.

### Cell killing versus nerve stimulation thresholds for uni- and bipolar pulses

As explained in the Introduction, a smaller ratio of ablation and excitation thresholds translates to a reduced span of stimulation outside of the ablation site. To identify the pulse duration and shape to minimize neuromuscular effects, we compared the strength-duration curves for cell death with our recent measurements of the peripheral nerve excitation thresholds^43^ (Fig. 9). We used the cell death thresholds for 300-pulse trains since they were smaller than for the shorter trains, meaning a smaller ratio to excitation thresholds. The nerve stimulation thresholds in Fig. 9 are for the most excitable fibers and using the electrode configuration that yields the lowest excitation thresholds, making it “the worst-case scenario” for neurostimulation.

All strength-duration curves expectedly climbed as pulse duration decreased (Fig. 9A). The nerve stimulation thresholds rose much faster (similar to findings with H-FIRE^17^), thereby decreasing the ratio of cell killing and excitation thresholds for shorter pulses (Fig. 9B). Nanosecond pulses showed a profound and unequivocal advantage over conventional 100-µs pulses: The ratio of cell killing and excitation thresholds dropped from as high as 500 for unipolar 100-µs pulses to 13 for unipolar 150-ns pulses. Engaging bipolar cancellation further decreased this ratio to only 4 for bipolar 150 ns pulses (Fig. 9B). Thus, bipolar nanosecond pulses are the best choice for the reduction of neuromuscular stimulation from PEF ablation.

**Figure 10:**
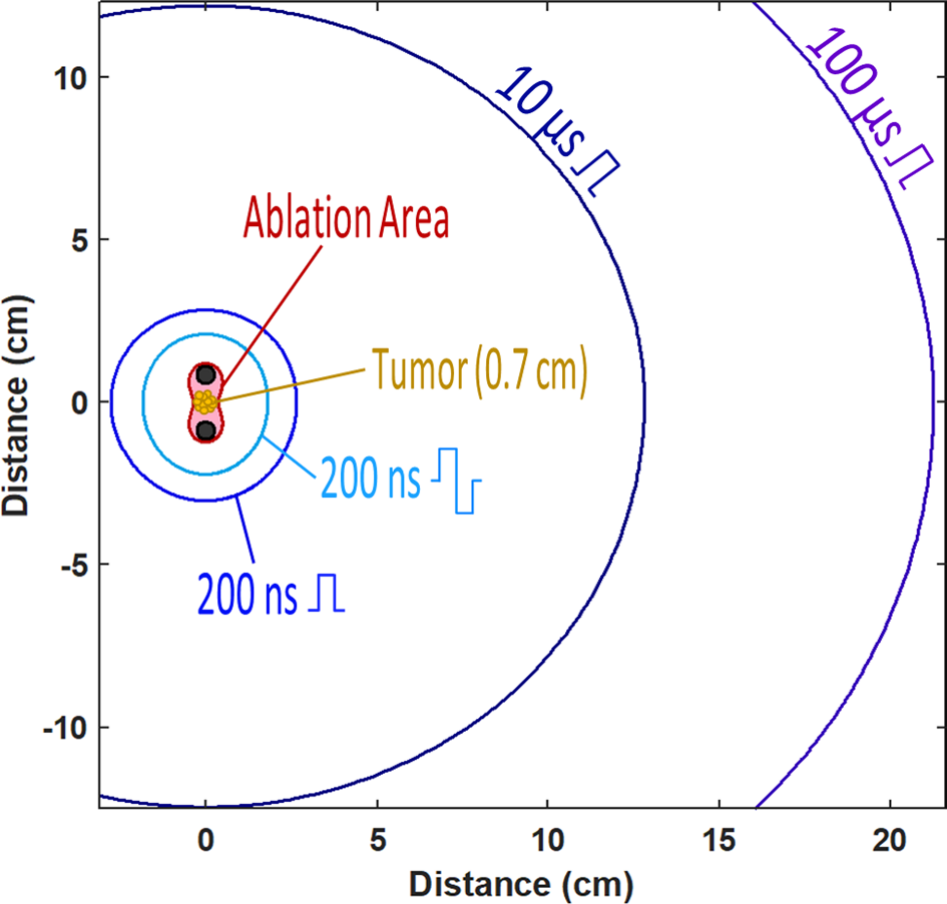
The projected areas of nerve stimulation when a hypothetical tumor is ablated by pulses of different shape and duration. This example uses data from Figs. 2F and 9B and assumes uniform dielectric properties of the tissue. The tumor is placed between two needle electrodes which pierce the tissue orthogonal to the plane shown in the figure. Pulse amplitude is set to produce the electric field sufficient to ablate the tumor (“Ablation Area”). The larger radii mark the projected distances at which the nerve stimulation is expected when the tumor is ablated by pulses of a specific shape and duration (legends). See Figs. 2, 9, and text for more details.

The stimulation boundaries for ablation with different pulse durations and shapes are illustrated by a hypothetical example in Fig. 10. Based on data in Fig. 9 and numerical simulations of the electric field distribution, we explored how far electrostimulation will reach from an ablation area. A tumor 0.7 cm in diameter was placed between two needle electrodes (0.5-cm diameter, 1.7-cm center-to-center distance) for ablation by PEF, and 300 pulses were delivered at the minimum amplitude needed for tumor ablation. Nerve stimulation occurred within a range of about 2 cm for bipolar 200-ns pulses, but as far as 20 cm away for unipolar 100-µs pulses.

### Absorbed dose for cell killing at different pulse durations

The reduction of the gap between cell killing and nerve excitation thresholds is not the only criterion to be considered when planning ablation treatments. Tissue heating by PEF is unavoidable but should be kept to minimum to ensure cell death from electroporative membrane damage and not from hyperthermia.

Thermal effects are proportional to the absorbed dose and are offset by heat dissipation, which depends on the total time of PEF treatment and the efficacy of heat exchange. A lower absorbed dose is desirable to reduce the potential thermal effects.

**Figure 11:**
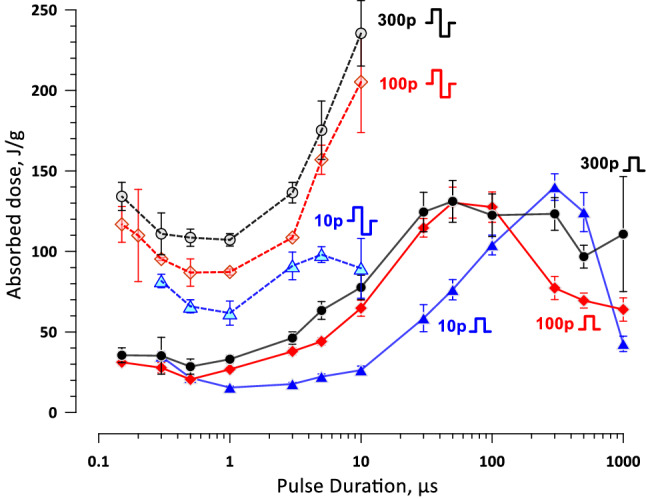
The absorbed dose, J/g, at the threshold for cell killing by uni- and bipolar pulses of different duration (solid and dashed lines, respectively). For bipolar pulses, the dose is plotted against the duration of one phase. The pulse shape and the number of pulses per train are labeled next to the respective curves (“10p”, “100p”, and “300p” stand for 10, 100, and 300 pulses per train respectively). Pulse repetition rate was 10 Hz. See text and Figs. 6 and 7 for more details.

We analyzed how the absorbed dose at the cell killing threshold changes with pulse duration for uni- and bipolar pulse trains (Fig. 11). The absorbed dose was calculated in a standard manner^63^. For all tested conditions, the absorbed dose was minimal at 0.5–1 µs pulse duration and increased for both shorter and longer pulses, in a complex manner. For most pulse durations, shorter PEF trains achieved cell killing at lower doses. Bipolar pulses expectedly required higher doses, because of both the higher thresholds and the additional energy deposited by the 2^nd^ phase of the pulse. Higher energy expenditure by the bipolar pulses is a factor to consider when choosing between the non-thermal cell killing, minimizing neuromuscular effects, and extending PEF treatment time to allow heat dissipation.

### The balance of reversible and irreversible electroporation at different pulse durations

Lower thresholds for reversible electroporation compared to cell death (irreversible electroporation) translate into a transient cell damage in the vicinity of the ablation area. Damaged membrane loses the resting membrane potential, eventually culminating in action potential firing in excitable cells ^19,20^.

**Figure 12:**
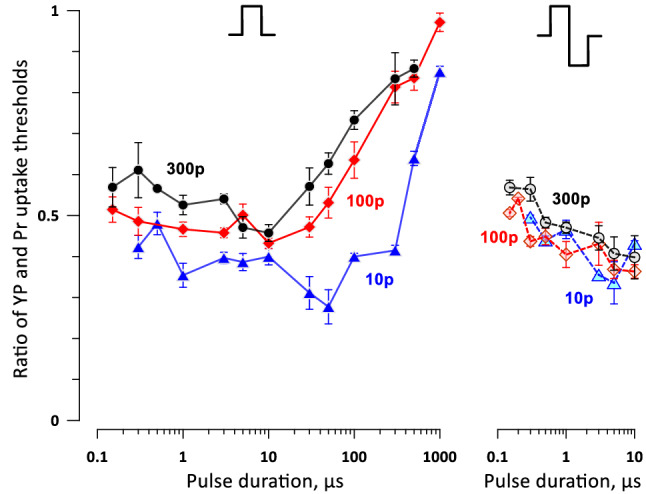
The ratios of electric field strengths thresholds for reversible and irreversible electroporation (cell death) at different pulse durations. For clarity, the ratios were plotted separately for trains of unipolar and bipolar pulses (left and right panels). Labels “10p”, “100p”, and “300p” stand for trains of 10, 100, and 300 pulses, respectively. See Fig. 11 and text for more details.

We evaluated the balance of reversible and irreversible electroporation by the ratio of the electric field thresholds for YP and Pr uptake (Fig. 12). The higher ratio (approaching 1) translates in a narrow edge of reversible electroporation outside of the cell death area and is preferable for ablation treatments. For trains of unipolar pulses (Fig. 12, left panel), the highest ratio was observed with the longest 0.5- and 1-ms pulses. The ratio gradually decreased to the minimum of 0.3–0.5 as the pulse duration decreased to 50 µs (10-pulse trains) or 10 µs (100- and 300-pulse trains), and slightly increased again as the pulses shortened into the nanosecond range. Longer 300-pulse trains had a somewhat higher ratio across most of the studied pulse durations. The balance of the reversible and irreversible electroporation thresholds was not significantly affected by the addition of the 2nd phase (Fig. 12, right panel).

For trains of 300 bipolar 150-ns pulses (the most promising parameters to reduce the neuromuscular effects, Figs. 9 and 10), the YP/Pr threshold ratio was between 0.5 and 0.6. This means the reversible electroporation stays within the calculated margins of nerve stimulation around the ablation area and should not add significantly to neuromuscular effects. However, these numbers may change and should be validated for specific cell and tissue types.

### Electroporation and cell killing by a series of nsPEF bursts: The effect of pulse repetition rate within the bursts

We showed previously that packing multiple nanosecond pulses into brief bursts evokes just a single action potential when the burst fits within the nerve refractory period ^43^. This approach is promising to reduce the number of neuromuscular responses to ablation treatments with multiple pulses. However, it is not known if nsPEF packing into high-rate bursts might compromise their ablation efficiency.

We measured the cell death and electroporation thresholds for one hundred of uni- and bipolar 600-ns pulses, which were arranged in 10 bursts of 10 pulses each. The frequency within the bursts was varied from 10 Hz to about 1 MHz, while the bursts were delivered at a constant rate of 1 Hz, to allow time for heat dissipation. This way, all PEF treatments took about 10 s, with the exception of 1 Hz pulses which were delivered as a single train lasting 100 s.

**Figure 13:**
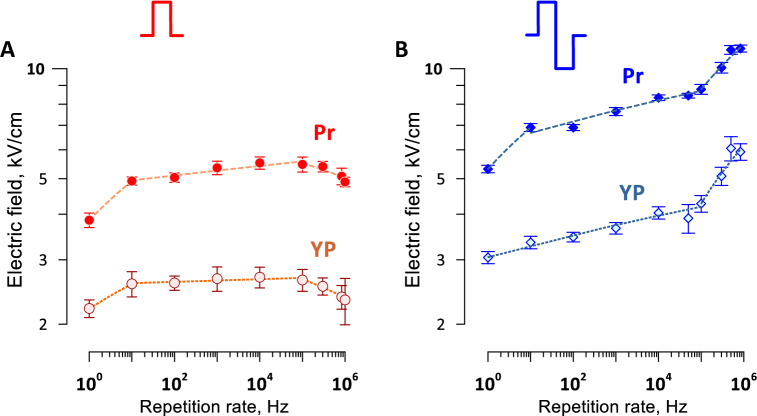
The effect of pulse repetition rate on the thresholds for electroporation (YP) and cell killing (Pr) by bursts of unipolar (**A**) and bipolar (**B**) pulses. Ten bursts of 10 pulses each were delivered at a constant rate of 1 Hz, while the repetition rate within the bursts was varied. At the lowest rate shown (10^0^ Hz), pulses were delivered as a single 100-pulse train. Pulse duration (**A**) and single phase duration (**B**) was 600 ns. Dashed lines are best fits using power function. Electroporation thresholds were measured from the borders of YO-PRO-1 (YP) dye uptake; the dye was added before the pulse treatment and removed 30 min after it. Electric field thresholds for cell death were measured from the borders of propidium (Pr) staining when the dye was added 2 h after the pulse treatment. See text for more details.

The repetition rate had weak if any effect on the efficiency of unipolar pulses (Fig. 13A). A small but significant reduction of the threshold was observed with the longest 100-s, 1-Hz treatments, which was most likely the result of electrosensitization^41,42^. Increasing the pulse frequency beyond 100 kHz also resulted in a modest reduction of the thresholds, which was far smaller than seen when all pulses were compressed into a single high-rate train^64–66^.

For bipolar pulses, the cell killing threshold also was the lowest for 100-s, 1-Hz treatments (Fig. 13B). Both the electroporation and cell killing thresholds increased slowly, by about 30% total, as the pulse frequency increased by four orders of magnitude (from 10 Hz to 100 kHz). Above the critical frequency of 100 kHz, both thresholds increased faster, by 30–50% as the pulse frequency increased by less than one order of magnitude. Apparently, fast charging and discharging of the membrane by bipolar pulses reduced the integral time when the transmembrane potential was above the electroporation threshold. It contrasted the reduction of threshold for unipolar pulses at short intervals which enabled the temporal summation of the induced membrane potential (Fig. 13A).

Overall, packing uni- and bipolar pulses into high-rate bursts had relatively minor effect on the death threshold, supporting the use of high-rate burst protocols for further reduction of neuromuscular effects of PEF ablation.

## Discussion

The experiments prove a strong and unambiguous advantage of ablation with nsPEF over “classic” IRE with 100-µs pulses for the reduction of neuromuscular side effects. Shortening pulses within the nanosecond range and delivering bipolar pulses reduces the stimulation span further, although not as profoundly (Figs. 9 and 10). A tenfold decrease of the stimulation radius from 100-µs pulses to bipolar 200-ns pulses (Fig. 10) translates into an impressive 1,000-fold reduction of the stimulated tissue volume. The numbers may differ for other cell and tissue types^49,67,68^, as well as for other types of nerve fibers, animal species, and in vivo treatments, but general trends how the thresholds change with pulse duration and shape will likely be universal. Our conclusions are also consistent with earlier theoretical and experimental reports that shortening PEF duration into the nanosecond range decreases neuromuscular response to PEF ablation^17,37,39,47,69,70^. Some of our experimental measurements have come in a remarkable agreement with earlier predictions that combined numerical models of nerve fibers and irreversible electroporation data^17,47^.

The best results in terms of the smallest ratio of excitation and ablation thresholds were achieved with the longest trains of 300 pulses. It should be kept in mind that a 300-pulse train at 10 Hz will cause 300 neuromuscular responses, which may be a less desirable outcome than fewer responses even if the stimulation spreads further away. A simple increase of the pulse repetition rate may result in prohibitively intense heating and the reduction of nerve excitation threshold (i.e., farther spread of excitation) at duty cycles above 0.1%^43^. Instead, nanosecond pulses can be delivered in brief bursts which do not exceed the nerve excitation refractory period. This approach enables the reduction of the number of neuromuscular responses (e.g., a tenfold reduction with 10 pulses per burst) while allowing ample time for heat dissipation between the bursts. Increasing the frequency of bipolar nsPEF up to 100 kHz within a burst attenuates their killing efficacy just modestly, by 10–20% (Fig. 13B). The temporal summation will concurrently decrease the nerve stimulation threshold by up to 1.6 times^48^, which will spread the stimulation a little further away. The optimal balance of ablation, stimulation, and heating will likely be achieved at intermediate repetition rates of 10–50 kHz and bursts of 10–30 bipolar pulses with 100–200 ns phase duration.

It is worth noting that bursts of unipolar nsPEF offer a smaller but comparable advantage in terms of the reduction of neuromuscular effects. At the same time, unipolar nsPEF achieve ablation at much lower absorbed doses than bipolar pulses (Fig. 11), making it easier to mitigate heating.

Unexpectedly, both uni- and bipolar nsPEF caused a larger fringe of reversible electroporation than pulses over 100 µs in duration (Fig. 12). While this is an undesirable feature for ablation, it is modest compared to the massive reduction of the neuromuscular stimulation volume when using nsPEF.

## Data Availability

All data generated or analyzed during this study are available from the corresponding author on reasonable request.
